# Hyperparathyroidism Two Years after Radioactive Iodine Therapy in an Adolescent Male

**DOI:** 10.1155/2014/163848

**Published:** 2014-01-30

**Authors:** Danielle L. Gomez, Dorothy I. Shulman

**Affiliations:** University of South Florida Diabetes Center, Department of Pediatrics, University of South Florida Morsani College of Medicine, 12901 Bruce B. Downs Boulevard, MDC 62, Tampa, FL 33612, USA

## Abstract

Primary hyperparathyroidism is a very rare complication following radioactive iodine therapy. There is typically a latency period of more than a decade following radiation exposure and, therefore, it is observed almost exclusively in adults. Consequently, pediatricians are not aware of the association. We present a case of primary hyperparathyroidism due to a solitary parathyroid adenoma occurring in an adolescent male two years following radioactive iodine treatment for papillary thyroid carcinoma. Periodic screening of serum calcium following ablative doses of radioactive iodine for thyroid cancer may be justified even in adolescents.

## 1. Introduction

Primary hyperparathyroidism (P-HPT) is uncommon in children and adolescents with an incidence of 2–5 in 100,000 [1]. Excess parathyroid hormone (PTH) secretion leads to hypercalcemia, hypophosphatemia, nephrocalcinosis, nephrolithiasis, and decreased bone density. Parathyroid adenoma is the most common cause of P-HPT in young patients, representing 65–90% of pediatric cases [1–4]. Multiglandular hyperplasia occurs in 16–27% of pediatric P-HPT, half of which can be attributable to multiple endocrine neoplasia (MEN)-I or MEN-II syndromes or familial non-MEN HPT [1, 2]. Due to its rarity in children, most of the literature on P-HPT in pediatric patients has been limited to case reports and small series [1–4].

Radioactive iodine treatment (RAI) is an accepted treatment for hyperthyroidism associated with Graves' disease and metastatic papillary thyroid carcinoma (PTC) following surgical resection in adults and children [5, 6]. Colaço et al. analyzed 47 patients who developed P-HPT following treatment with RAI for benign and malignant disease [7]. A female predominance was observed (89.4% women), with P-HPT occurring at a mean age of 59.4 ± 13.5 years. The average latency period to the development of P-HPT after RAI was 13.5 ± 9.1 years. In this review, two patients with Graves' disease were less than 18 years old (5 and 11 years) at the time of RAI, and time to development of P-HPT was 31 and 22 years, respectively [8]. Surgical pathology in these individuals revealed 4-gland hyperplasia in one and an adenoma in the other. We report a case of a 17-year-old male who presented with hypercalcemia 2 years following RAI treatment for PTC. To our knowledge, this is the first patient reported with this presumed complication of RAI exposure presenting in the pediatric age range.

## 2. Case Presentation

**Figure 1:**
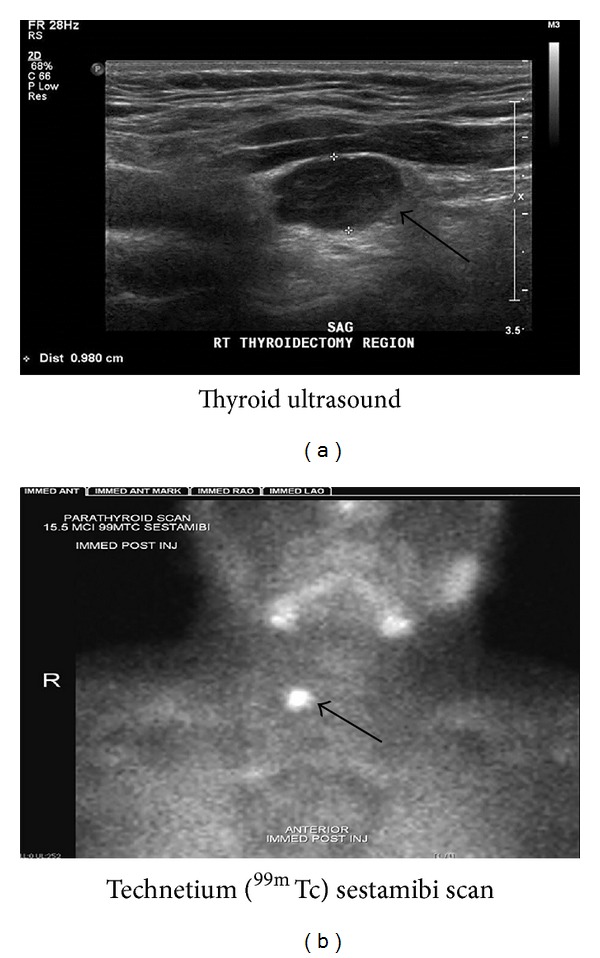
Ultrasound (a) and technetium (^99m^Tc) sestamibi scan (b) of nonpalpable 1 cm nodule in right neck prior to removal of parathyroid adenoma.

A 17-year-old male presented to the pediatric emergency room with severe headaches. Past medical history was significant for congenital hydrocephalus requiring ventriculoperitoneal shunting and a congenital vein of Galen aneurysm treated with metal coil embolization in infancy at another institution. In the course of diagnosis and management of the hydrocephalus during childhood, he had had 14 computerized tomographic (CT) studies of the brain. At 15 years, he was diagnosed with PTC metastatic to local lymph nodes in the right neck that was treated with a total thyroidectomy, neck dissection, RAI therapy, and L-thyroxine suppression. There were no postoperative complications. He received two doses of RAI a year apart, a total of 263 millicuries (mCi). Serum thyroglobulin (Tg) level off thyroxine prior to second RAI treatment was 6.4 ng/mL, while serum thyroid-stimulating hormone (TSH) was 81 *μ* IU/mL, suggesting some residual thyroid-like tissue. Post-I131 treatment total body scan obtained 5 days after the second therapy dose showed uptake in right neck, interpreted as probable residual thyroid cancer. Three months after the second RAI dose, he developed severe headaches and was found to have an intracranial thrombus of the transverse sinus. At this time, serum calcium was 10 mg/dL and Tg concentration on thyroxine therapy was <1 ng/mL. He was treated with anticoagulation for one year. At both 7 and 11 months following the second RAI treatment, surveillance neck ultrasounds revealed the stable appearance of a 1 cm nodule in the right thyroid bed that was not palpable on physical examination. Fine needle aspirate was planned after stopping anticoagulation therapy. One week after anticoagulation therapy was discontinued, headaches acutely returned prompting the emergency room visit. Brain CT showed progressive, nonocclusive thrombosis of the transverse sinus. Laboratory studies performed during the emergency room visit identified hypercalcemia (13.1 mg/dL; normal range 8.9–10.7) and hypophosphatemia (2 mg/dL; normal range 3.0–5.2), with a concomitant intact PTH of 154 pg/mL (normal range 9–69). Anticoagulant therapy was resumed. Neck ultrasound and technetium (^99m^Tc) sestamibi scan were consistent with a hyperfunctioning right parathyroid adenoma (Figure 1). Prior to proceeding with surgical excision, recombinant thyrotropin-stimulated serum Tg concentration was <1 ng/mL and total body 4 mCi I 131 scan was negative. The patient underwent removal of a 1.5 cm right parathyroid adenoma with benign histology. Fifteen hours postoperatively, serum PTH and calcium were <3 pg/mL and 9.3 mg/dL, respectively. The patient was transiently treated with calcium supplementation and remained eucalcemic 2 years following surgery. Follow-up neck ultrasound at 2 years reveals no nodules.

## 3. Discussion

Hyperparathyroidism was first reported following external beam radiation in 1975 and has subsequently been described following head and neck radiation for benign and malignant conditions and among atomic bomb survivors of Hiroshima [9–11]. Cohen et al. evaluated a cohort of 4297 patients who received radiation to the tonsils before the age of 16 years and were followed prospectively. The incidence of clinical hyperparathyroidism was increased approximately 2.5-fold over the general population and the latency period was 20 to 46 years [12]. McMullen et al. recently reported the incidental finding of unsuspected hyperparathyroidism largely due to adenoma in 10% of 53 patients referred for nodular thyroid disease following radiation for childhood cancer [13]. Latency period was 15 to 34 years. Estimated radiation exposure to the neck was 90–1320 centigray (cGy). In the adult literature, hyperparathyroidism is a rare occurrence following RAI treatment, which is observed mostly in females and with an average latency period of 15–20 years following RAI exposure [7]. Hyperparathyroidism following RAI in pediatric patients treated for Graves' disease is reported, but typically occurs during adulthood, more than a decade later [8, 14]. We found a single report of hyperparathyroidism occurring in a pediatric thyroid cancer patient at the age of 22 years treated with 103 mCi of RAI at age 9 years [15]. Hypoparathyroidism has also been reported following RAI and is usually transient [16–18].

Estimated dose to the parathyroids following treatment of Graves' disease (doses 2–38 mCi) is 140 to 750 cGy [19]. Our patient received a total RAI dose of 263 mci, 10-fold higher than that typically administered for Graves' disease. The incidence of hyperparathyroidism has been suggested to increase with the dose of radiation exposure [20, 21] and younger age at exposure [15]. Studies of radiation-exposed populations have demonstrated a much greater sensitivity to radiation in children compared with adults, presumably in part due to greater mutagenic effects of radiation in growing children's tissues, which are replicating at a faster rate [22]. Doses as small as 50–100 mGy have been associated with an increased risk of thyroid malignancy in children, with a linear dose-response up to about 10–20 Gy after which the risk appears to level off [23–25]. This excess risk persists for at least four decades after exposure [23]. Our patient had numerous CT scans as a young child, estimated to yield a cumulative exposure of 20–40 cGy to the head in addition to that from RAI [26]. The contribution of radiation exposure from early CT scans to our patient's thyroid cancer and/or hyperparathyroidism is unclear.

Pediatricians are well aware that hypoparathyroidism is a known complication of thyroidectomy and thyroid cancer surgery. In patients who do not have postoperative hypocalcemia, calcium levels may not subsequently be monitored. Our patient demonstrates that, while rare, hyperparathyroidism may occur following a short latency period after RAI. We recommend that serum calcium levels be included in routine yearly surveillance of these patients, particularly in those with a history of additional radiation exposure.
